# Explainable Deep Learning for Breast Lesion Classification in Digital and Contrast-Enhanced Mammography

**DOI:** 10.3390/diagnostics15243143

**Published:** 2025-12-10

**Authors:** Samara Acosta-Jiménez, Miguel M. Mendoza-Mendoza, Carlos E. Galván-Tejada, José M. Celaya-Padilla, Jorge I. Galván-Tejada, Manuel A. Soto-Murillo

**Affiliations:** Unidad Académica de Ingeniería Eléctrica, Universidad Autónoma de Zacatecas, Jardín Juárez 147, Centro, Zacatecas 98000, Mexico; samaracosta@uaz.edu.mx (S.A.-J.); mauricio.mendoza@uaz.edu.mx (M.M.M.-M.); jose.celaya@uaz.edu.mx (J.M.C.-P.); gatejo@uaz.edu.mx (J.I.G.-T.); alejandro.somu@uaz.edu.mx (M.A.S.-M.)

**Keywords:** breast cancer, digital mammography, contrast-enhanced spectral mammography, CNNs, explainable AI

## Abstract

**Background**: Artificial intelligence (AI) emerges as a powerful tool to assist breast cancer screening; however, its integration into different mammographic modalities remains insufficiently explored. Digital Mammography (DM) is widely accessible but presents limitations in dense breast tissue, whereas Contrast-Enhanced Spectral Mammography (CESM) provides functional information that enhances lesion visualization. Understanding how deep learning models behave across these modalities, and determining whether their decision-making patterns remain consistent, is essential for equitable clinical adoption. **Methods**: This study evaluates three convolutional neural network (CNN) architectures, ResNet-18, DenseNet-121, and EfficientNet-B0, for binary classification of breast lesions using DM and CESM images from the public CDD-CESM dataset (2006 images, three diagnostic classes). The models are trained separately on DM and CESM using three classification tasks: Normal vs. Benign, Benign vs. Malignant, and Normal vs. Malignant. A 3-fold cross-validation scheme and an independent test set are employed. Training uses transfer learning with ImageNet weights, weighted binary cross-entropy (BCE) loss, and SHapley Additive exPlanations (SHAP) analysis to visualize pixel-level relevance of model decisions. **Results**: CESM yields higher performance in the Normal vs. Benign and Benign vs. Malignant tasks, whereas DM achieves the highest discriminative ability in the Normal vs. Malignant comparison (EfficientNet-B0: AUC = 97%, Accuracy = 93.15%), surpassing the corresponding CESM results (AUC = 93%, Accuracy = 85.66%). SHAP attribution maps reveal anatomically coherent decision patterns in both modalities, with CESM producing sharper and more localized relevance regions due to contrast uptake, while DM exhibits broader yet spatially aligned attention. Across architectures, EfficientNet-B0 demonstrates the most stable performance and interpretability. **Conclusions**: CESM enhances subtle lesion discrimination through functional contrast, whereas DM, despite its simpler acquisition and wider availability, provides highly accurate and explainable outcomes when combined with modern CNNs. The consistent SHAP-based relevance observed across modalities indicates that both preserve clinically meaningful information. To the best of our knowledge, this study is the first to directly compare DM and CESM under identical preprocessing, training, and evaluation conditions using explainable deep learning models.

## 1. Introduction

Breast cancer is the most frequently diagnosed malignancy and a leading cause of cancer-related death among women worldwide. According to the World Health Organization, an estimated 2.3 million women are diagnosed with breast cancer and 670,000 deaths occur globally in 2022, underscoring the need for effective early detection strategies [1,2]. Prognosis is strongly associated with stage at diagnosis, making timely screening a cornerstone of breast cancer control.

Digital Mammography (DM) remains the gold standard for population-based screening owing to its availability, reproducibility, and relatively low cost [3]. However, its diagnostic performance declines in women with dense breast tissue, where tissue overlap may obscure lesions or generate false-positive findings, leading to unnecessary biopsies or missed diagnoses [4,5,6]. These limitations have stimulated interest in imaging techniques that combine morphological and functional information, particularly contrast-enhanced modalities such as Contrast-Enhanced Spectral Mammography (CESM) [7,8].

CESM has emerged as a complementary modality that augments conventional X-ray imaging with iodine-based contrast, enabling visualization of hypervascular regions commonly associated with malignancy. State-of-the-art reviews report improved lesion conspicuity and diagnostic confidence with CESM, highlighting its expanding roles in diagnosis and procedural guidance [7,9,10,11,12]. Accumulating evidence demonstrates that CESM provides diagnostic performance comparable to breast magnetic resonance imaging (MRI). Several comparative studies report that CESM achieves sensitivities equivalent to MRI while offering practical advantages such as shorter examination time [13,14]. A 2022 study additionally describes slightly higher overall accuracy and specificity for CESM in the evaluation of multifocal and multicentric breast cancer [15]. Likewise, a 2020 meta-analysis finds pooled sensitivities of approximately 97% for both modalities, with a diagnostic odds ratio favoring CESM [16]. Moreover, a systematic review encompassing 19 studies confirms consistently high sensitivities (≈97%) for both modalities and, in several instances, superior specificity with CESM [17]. Collectively, this body of evidence supports CESM as a robust and more accessible alternative to MRI for breast cancer detection, particularly in healthcare settings with limited MRI availability. Despite these advantages, CESM requires intravenous iodinated contrast and dedicated workflows, which may limit widespread implementation, particularly in resource-constrained health systems. Furthermore, iodinated contrast introduces risks of hypersensitivity reactions and kidney-related complications, prompting professional guidance documents to provide patient-selection and management recommendations [18,19].

These clinical and operational constraints underscore the need for complementary, non–contrast-dependent strategies to strengthen diagnostic performance and support decision-making across breast imaging modalities. Although CESM provides valuable functional enhancement, its reliance on contrast agents and specialized workflows continues to restrict widespread adoption, especially in resource-limited settings. In parallel, artificial intelligence (AI) has emerged as a powerful tool to complement imaging-based decision-making. Deep learning (DL) approaches, particularly convolutional neural networks (CNNs), demonstrate strong performance in mammography for detection and classification tasks [20,21,22,23]. However, most studies focus on a single imaging modality, and comparative evidence across DM and CESM remains scarce. Assessing whether both modalities reveal consistent visual cues offers valuable insights into the robustness and generalizability of AI-based breast lesion assessment.

The recent literature highlights the importance of explainable AI (XAI) in breast imaging, particularly for understanding modality-specific errors and identifying features that influence model predictions. Shifa et al. (2025) emphasize that because diagnostic decisions have major implications for patient outcomes, understanding the rationale behind AI predictions is essential [24]. Similarly, a 2023 systematic review by Gurmessa et al. reports that XAI not only enhances accuracy and reduces human error but also addresses key ethical challenges by promoting transparency, accountability, robustness, and the right to information in machine learning–based decision making [25]. Despite these advances, only a limited number of studies extend XAI analyses to both DM and CESM, leaving unresolved whether their diagnostic patterns align or diverge when evaluated using deep learning models.

This limitation is further amplified by recent trends in CESM-focused AI research, which largely remain modality-specific. Most recent AI studies involving CESM restrict analyses to this modality alone. Dominique et al. (2022), for example, propose a CNN-based CESM classifier but do not evaluate its behavior on DM images [26]. Likewise, Jailin et al. (2023) develop a CESM-based multimodal CAD framework without performing direct cross-modality comparisons [27]. Because DM and CESM differ in acquisition physics, contrast enhancement patterns, and tissue representation, the absence of harmonized cross-modality evaluations limits our understanding of how AI models generalize across imaging techniques. This gap underscores the need for systematic comparisons under identical preprocessing, training, and evaluation conditions.

DL research in mammography advances rapidly with the emergence of transformer-based and hybrid attention architectures. Studies published between 2022 and 2024 show that these models outperform traditional CNNs in lesion localization, breast density prediction, and malignancy classification [28,29,30]. Chang et al. (2025) report that deep-learning systems trained on large, multi-institutional datasets achieve screening performance approaching that of radiologists [30]. Brahmareddy et al. (2025) further demonstrate that a multimodal, multitask hybrid CNN–Transformer framework enhances breast cancer diagnosis by jointly modeling spatial, temporal, and clinical information, outperforming state-of-the-art baselines in both subtype classification and stage prediction while improving interpretability through integrated explainability modules [28]. Despite these advances, most investigations remain modality-specific, underscoring the need to examine whether consistent diagnostic patterns emerge across DM and CESM. Collectively, these observations highlight the importance of developing a unified analytical framework capable of assessing modality-consistent diagnostic patterns using contemporary deep learning and explainability techniques.

The present study investigates whether similar visual diagnostic patterns are identified in DM and CESM using explainable deep learning models. In this context, SHapley Additive exPlanations (SHAP) provides pixel-wise attribution scores that quantify how each image region contributes to a prediction, thereby making CNN decisions more transparent and clinically interpretable [31]. Three CNN architectures, ResNet-18, DenseNet-121, and EfficientNet-B, are evaluated across three clinically relevant binary tasks (Normal vs. Benign, Benign vs. Malignant, and Normal vs. Malignant). To elucidate model behavior, SHAP is applied to generate pixel-level attribution maps in both modalities. The comparison of these explanations between DM and CESM enables identification of consistent visual cues that may support lesion characterization even when CESM is unavailable, thus promoting equitable access to AI-assisted, high-quality breast imaging in resource-limited healthcare settings.

This article is an extended version of the conference paper “Interpretable Deep Learning for Breast Lesion Classification: A SHAP-Based Comparison of CESM and Digital Mammography,” presented at AHTBE 2025 (Paper ID: AH1558).

## 2. Materials and Methods

**Figure 1:**
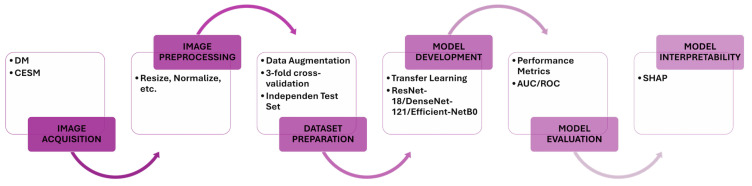
Proposed workflow. The diagram summarizes the key stages of this study, including image acquisition, preprocessing, data augmentation, model training, cross-validation, independent test evaluation, and SHAP-based interpretability. Identical procedures are applied to DM and CESM to ensure harmonized and comparable analyses.

This study evaluates the classification and interpretability performance of CNNs for breast lesion analysis using two mammographic modalities: DM and CESM. Each model is independently trained and evaluated on both modalities using identical preprocessing and hyperparameter configurations to ensure a fair comparison. The experimental workflow comprises image acquisition, preprocessing, data augmentation, model training, cross-validation, independent test evaluation, and post hoc interpretability analysis using SHAP. An overview of this pipeline is illustrated in Figure 1, which summarizes the sequential stages of data handling, model development, and interpretability assessment.

### 2.1. Image Acquisition

The experiments use the publicly available Contrast-Enhanced Digital Database for CESM (CDD-CESM) dataset hosted on The Cancer Imaging Archive (TCIA) [32]. The dataset contains 2006 mammographic images from 326 female patients aged 18 to 90 years. Each patient undergoes both low-energy (DM-equivalent) and contrast-enhanced (CESM) acquisitions obtained with GE Senographe DS and Hologic Selenia Dimensions systems. Craniocaudal (CC) and mediolateral oblique (MLO) views are available for most cases, although not all patients present both projections. All images are provided in JPEG format with three color channels and an average resolution of 2355 × 1315 pixels. Lesions are categorized as normal, benign, or malignant based on histopathological confirmation or expert annotation. Table 1 summarizes the distribution of images across diagnostic categories and modalities.

No additional exclusion criteria are applied. The dataset is used as released, with some patients providing both CC and MLO views and others providing only a single projection.

### 2.2. Image Preprocessing

Preprocessing steps are summarized in Table 2. Data augmentation is applied only to training images, including random brightness and contrast adjustments, horizontal and vertical flips, and in-plane rotation, to enhance model robustness and mitigate overfitting. All images are resized to 224 × 224 pixels and normalized using ImageNet statistics. Validation and test sets undergo only resizing and normalization to ensure consistent evaluation. The target resolution of 224×224 pixels is selected to match the default input size of ResNet-18, DenseNet-121, and EfficientNet-B0, ensuring compatibility with their ImageNet pre-trained weights. Images are normalized using the ImageNet mean and standard deviation, a standard transfer-learning practice that stabilizes training and improves convergence.

### 2.3. Model Architecture and Training

The three architectures used in this study, ResNet-18, DenseNet-121, and EfficientNet-B0, are selected to represent complementary convolutional design strategies. ResNet-18 employs residual connections to stabilize optimization [33]; DenseNet-121 leverages dense feature reuse to enhance gradient flow and parameter efficiency [34]; and EfficientNet-B0 applies compound scaling to balance depth, width, and resolution with high computational efficiency [35]. This selection provides a diverse representational spectrum for evaluating modality-dependent performance.

All models use ImageNet pre-trained weights and are fine-tuned for binary classification. The final fully connected layer is replaced with a single linear neuron that produces one output logit per image, followed by a sigmoid activation during inference. Each architecture is independently trained for every binary task and for each modality (DM and CESM) to ensure a fair comparison. Table 3 details the preprocessing steps implemented.

The dataset is split at the patient level into 80% for training and 20% for testing. Within the training portion, a threefold cross-validation (k=3) procedure is applied to estimate model generalization, where each fold is trained on two subsets and validated on the remaining one. Threefold cross-validation is selected to balance robustness and computational cost. Higher *k* values would leave too few samples per fold given the limited number of benign and malignant cases.

Class imbalance is handled using inverse-frequency weighting in the BCE loss, defined as follows:

$$w_{\mathrm{pos}}=\frac{N_{\mathrm{neg}}}{N_{\mathrm{pos}}},$$

with $w_{\mathrm{neg}}=1.0$. The weights are recomputed for every fold based on the corresponding sample counts.

To ensure clinically meaningful evaluation, this study defines three binary classification tasks: Normal vs. Benign, Benign vs. Malignant, and Normal vs. Malignant. These tasks reflect key diagnostic decision points in breast imaging. Normal vs. Benign evaluates the model’s ability to distinguish subtle non-malignant findings from healthy tissue, whereas Benign vs. Malignant targets the most challenging classification step and directly informs biopsy decisions. Normal vs. Malignant aligns with screening practice by separating healthy tissue from clearly suspicious lesions. Together, these tasks capture progressively complex diagnostic boundaries and enable a comprehensive assessment of model behavior.

### 2.4. Evaluation Metrics

Model performance is assessed using accuracy, precision, recall (sensitivity), F1-score, and the area under the receiver operating characteristic curve (AUC-ROC) [36]. All metrics are computed during cross-validation and final testing to ensure consistent evaluation.

Accuracy quantifies the overall proportion of correctly classified samples. Precision measures the proportion of true positives among all positive predictions. Recall captures the proportion of correctly identified positive cases. The F1-score provides a harmonic balance between precision and recall, particularly relevant in class-imbalanced tasks. The AUC-ROC quantifies the model’s ability to discriminate between classes across varying decision thresholds.

(1) $$\mathrm{Accuracy}=\frac{TP+TN}{TP+TN+FP+FN}$$

(2) $$\mathrm{Precision}=\frac{TP}{TP+FP}$$

(3) $$\mathrm{Recall}=\frac{TP}{TP+FN}$$

(4) $$F1-\mathrm{score}=2\times \frac{\mathrm{Precision}\times \mathrm{Recall}}{\mathrm{Precision}+\mathrm{Recall}}$$

All analyses and data visualizations are performed using Python (version 3.9.6). Key libraries include NumPy (version 1.26.4) [37], SciPy (version 1.13.1) [38], Matplotlib (version 3.9.0) [39], PyTorch (version 2.3.1) [40], SHAP (version 0.45.1) [31], and scikit-learn (version 1.5.0) [41].

### 2.5. Model Interpretability

Model interpretability is assessed using SHAP [31], a cooperative game–theoretic framework that assigns an importance value to each input feature based on its contribution to the model output. The GradientExplainer implementation is employed to estimate pixel-wise attributions from model gradients. SHAP values are computed for each test image using the full training split as background, ensuring that attributions are referenced to the same feature distribution used during model optimization. Positive SHAP values indicate regions that support the predicted class, whereas negative values highlight inhibitory areas. These attributions are visualized as heatmaps superimposed on the original mammograms to facilitate anatomical interpretation. For paired DM–CESM cases, SHAP maps are compared to determine whether both modalities emphasize similar lesion-related or anatomical regions across diagnostic classes.

The GradientExplainer receives three inputs: the trained CNN model, the training split used as background, and the test image being explained. Using a consistent background distribution promotes stable and clinically meaningful attributions. Reproducibility is ensured by fixing random seeds, applying identical preprocessing across splits, and maintaining consistent sample ordering when constructing the background set.

## 3. Results

### 3.1. Model Performance

Across both modalities (DM and CESM), EfficientNet-B0 consistently outperforms ResNet-18 and DenseNet-121, with the largest gains observed in the Normal vs. Malignant comparison. Models are evaluated using accuracy, precision, recall, F1-score, and AUC, and the results for each modality are summarized in Table 4 and Table 5.

#### 3.1.1. Digital Mammography

In the DM modality, all convolutional models achieve comparable yet distinct performances across the three binary classification tasks, yielding stable and interpretable results, as summarized in Table 4.

Regarding discriminatory ability, DenseNet-121 reports the lowest AUC values across the three tasks, achieving 44.88% for Normal vs. Benign, 65.50% for Benign vs. Malignant, and 75.46% for Normal vs. Malignant. ResNet-18 improves these values, reaching 54.44%, 58.50%, and 77.51%, respectively. EfficientNet-B0 provides the strongest overall discrimination, with AUC scores of 57.66%, 80.75%, and 97.33%. These results highlight the superior representational capacity of EfficientNet-B0, particularly in the Normal vs. Malignant task, where it achieves near-perfect separability.

A similar trend is observed for accuracy. DenseNet-121 attains accuracies of 37.88%, 57.89%, and 68.49% across the three tasks, while ResNet-18 achieves 50.00%, 49.12%, and 67.13%, respectively. EfficientNet-B0 again reports the highest performance in two of the three comparisons, reaching 54.55% in Normal vs. Benign, 63.16% in Benign vs. Malignant, and 93.15% in Normal vs. Malignant.

Precision results further emphasize the advantage of EfficientNet-B0, which obtains 43.59%, 73.91%, and 93.54% across the three tasks. In contrast, ResNet-18 reaches 38.89%, 56.52%, and 63.33%, while DenseNet-121 reports 30.00%, 65.38%, and 66.67%. These findings indicate that EfficientNet-B0 produces the most reliable positive predictions, especially in Normal vs. Malignant cases.

Recall and F1-scores complement these trends by illustrating the balance between correct positive identification and false-negative control. DenseNet-121 records F1-scores of 36.92%, 58.62%, and 61.02%, while ResNet-18 achieves slightly higher balance with 45.90%, 47.27%, and 61.29%. EfficientNet-B0 yields the strongest F1-scores, reaching 53.13%, 61.82%, and 92.06%.

Overall, the three architectures demonstrate consistent performance for DM classification; however, EfficientNet-B0 provides the most favorable results across AUC, precision, and accuracy, particularly for the Normal vs. Malignant task, which represents the most clinically relevant and challenging comparison. ResNet-18 offers more stable recall and F1 outcomes in the more balanced tasks, reflecting a conservative but resilient prediction profile.

**Figure 2:**
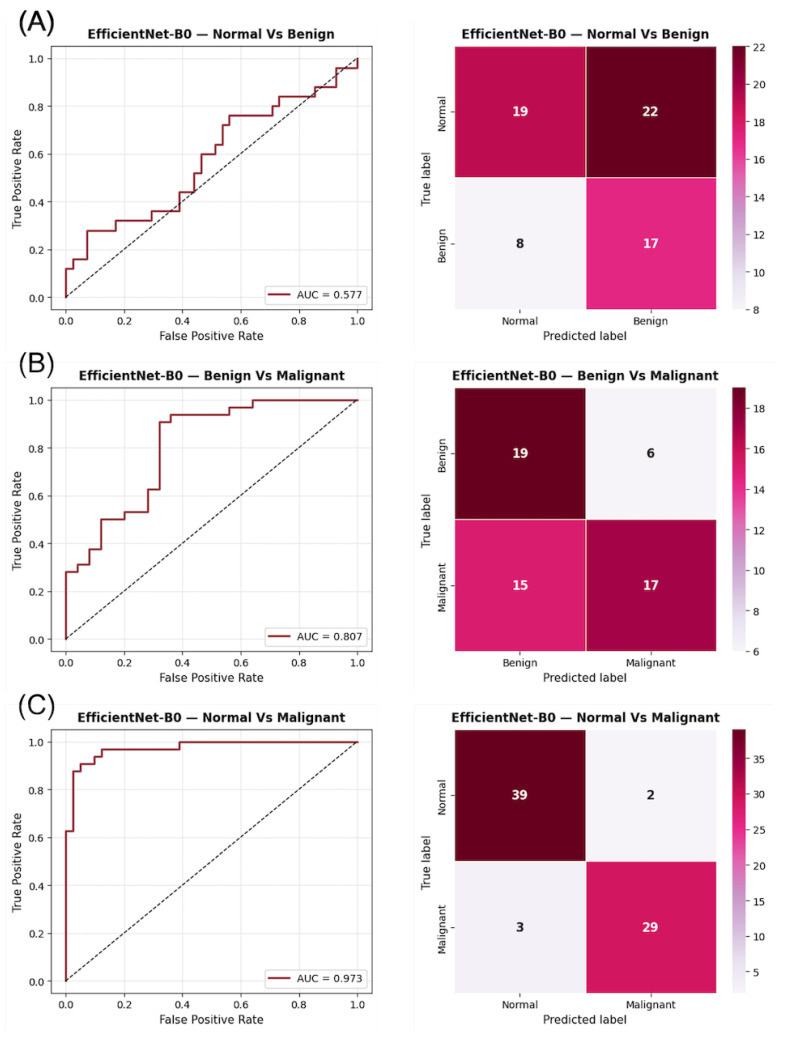
ROC curves and confusion matrices for EfficientNet-B0 using DM images in the three binary classification tasks: (**A**) Normal vs. Benign, (**B**) Benign vs. Malignant, and (**C**) Normal vs. Malignant. The AUC values indicate the discriminatory capacity for each task, while the confusion matrices summarize the prediction distribution for each category.

Visual inspection of the confusion matrices in Figure 2 reveals a consistent trend across the three binary tasks. In the Normal vs. Benign comparison, the classifier exhibits a near-random distribution of predictions, consistent with the AUC values close to 0.5, indicating a lack of discriminative learning between non-malignant categories. In contrast, the Benign vs. Malignant and Normal vs. Malignant matrices show stronger diagonal dominance, reflecting improved model certainty and clearer class separation. The ROC curves corroborate these observations, displaying steeper slopes and higher AUCs in tasks involving malignant lesions, which highlights the model’s increased sensitivity to cancer-related patterns.

#### 3.1.2. Contrast-Enhanced Spectral Mammography

In the CESM modality, all models achieve high and consistent performance across the three binary classification tasks, as summarized in Table 5. For the AUC metric, DenseNet-121 reports values of 55.90% for Normal vs. Benign, 78.55% for Benign vs. Malignant, and 93.87% for Normal vs. Malignant. ResNet-18 attains comparable results with AUCs of 62.05%, 72.00%, and 91.28%, respectively, while EfficientNet-B0 reaches 84.68%, 74.30%, and 93.27%. These findings confirm strong discriminatory ability for all architectures, particularly in the Normal vs. Malignant task, where AUC values exceed 90% across models.

Accuracy results follow a similar trend. DenseNet-121 achieves accuracies of 60.61%, 70.69%, and 89.19% across the three tasks, while ResNet-18 reports 59.09%, 65.52%, and 86.46%. EfficientNet-B0 obtains the highest accuracy in two of the three comparisons, reaching 77.27% for Normal vs. Benign, 72.41% for Benign vs. Malignant, and 87.88% for Normal vs. Malignant. These results underscore the stability of EfficientNet-B0, particularly in the Normal vs. Benign comparison, where it outperforms the other networks by a substantial margin.

Precision values further highlight model performance. DenseNet-121 achieves 47.83%, 75.00%, and 90.32%, while ResNet-18 reaches 47.37%, 72.41%, and 84.85%. EfficientNet-B0 again demonstrates superior precision, obtaining 70.83%, 81.48%, and 83.33%, respectively, indicating higher reliability in correctly identifying positive CESM cases.

Recall and F1-scores reinforce these observations. DenseNet-121 reports recall values of 44.00%, 72.73%, and 84.85%, while ResNet-18 achieves 72.00%, 63.64%, and 84.85%. EfficientNet-B0 provides the most balanced sensitivity, with recall values of 68.00%, 66.67%, and 90.91%. The F1-scores reflect these patterns: DenseNet-121 obtains 45.83%, 73.85%, and 87.50%; ResNet-18 reaches 57.14%, 67.74%, and 84.85%; and EfficientNet-B0 achieves 69.39%, 73.33%, and 86.96%. These results illustrate complementary strengths: EfficientNet-B0 favors precision with balanced sensitivity, whereas DenseNet-121 excels in overall discriminatory capacity.

Overall, all architectures demonstrate robust and stable CESM classification performance, with AUC values consistently above 70% and exceeding 90% in the Normal vs. Malignant task. EfficientNet-B0 provides the strongest overall accuracy and precision, while DenseNet-121 delivers slightly higher AUC and F1 performance in the more challenging comparisons. This consistent performance across models highlights the enhanced discriminative power of the CESM modality relative to DM, confirming its effectiveness in improving lesion classification through functional contrast information.

**Figure 3:**
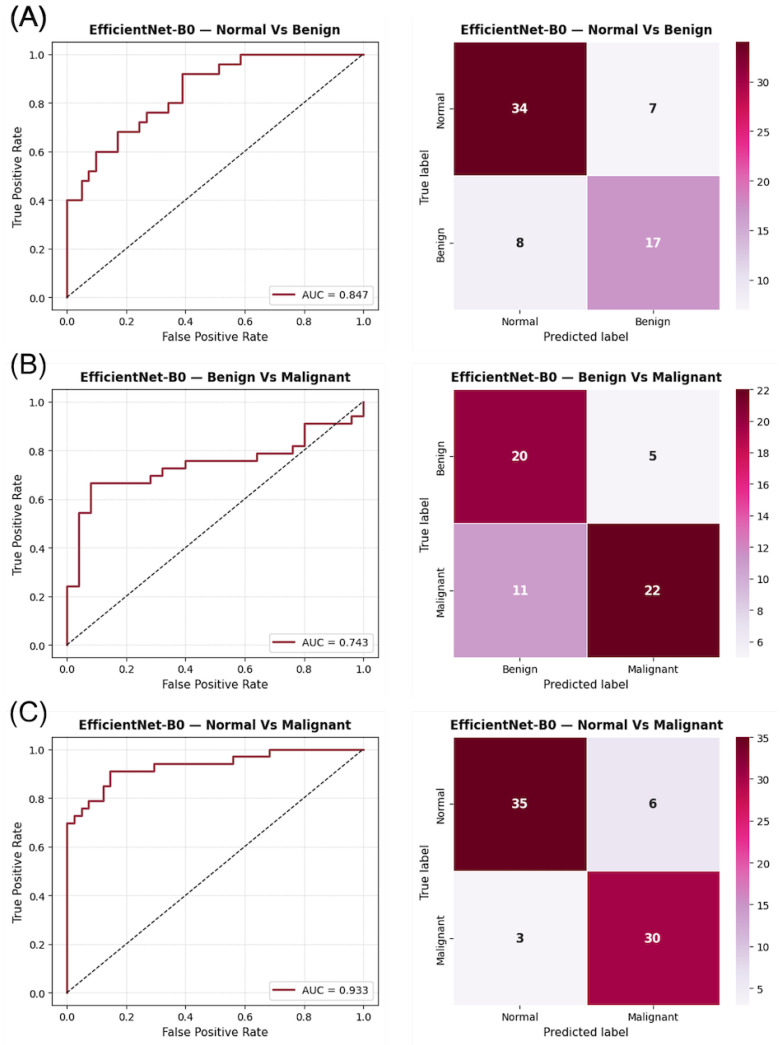
ROC curves and confusion matrices for EfficientNet-B0 using CESM images in the three binary classification tasks: (**A**) Normal vs. Benign, (**B**) Benign vs. Malignant, and (**C**) Normal vs. Malignant. The AUC values indicate the discriminatory capacity for each task, while the confusion matrices illustrate the prediction distribution for each category.

As shown in Figure 3, the confusion matrices for CESM indicate a more confident classification behavior, with fewer misclassifications along the off-diagonal positions compared to DM. The ROC curves exhibit sharper rises and greater AUC separation across all tasks, confirming the improved discriminative power of CESM. Notably, the Normal vs. Malignant comparison displays an almost perfectly diagonal distribution, illustrating the model’s ability to accurately identify malignant lesions with minimal false negatives. These graphical results support the quantitative improvements observed in CESM and provide visual confirmation of its enhanced lesion separability through functional contrast enhancement.

Overall, the comparison across architectures and modalities highlights the superior discriminative power of CESM and the strong generalization ability of EfficientNet-B0. The highest AUC values consistently appear in the Normal vs. Malignant task for both modalities, reflecting the clear morphological contrast between healthy tissue and overt malignancy. In contrast, the Benign vs. Malignant task yields the lowest AUCs, a well-known challenge arising from subtle benign–malignant differences and tissue overlap in mammography. CESM achieves higher AUCs in the Normal vs. Benign and Benign vs. Malignant tasks due to iodine-based contrast enhancement, which increases lesion conspicuity and highlights vascular features. However, DM slightly outperforms CESM in the Normal vs. Malignant comparison, indicating that structural and textural cues alone are sufficient for reliable separation in this less ambiguous diagnostic boundary. These modality-specific trends underscore the complementary value of DM and CESM. Importantly, the lower AUC and F1 values in the Benign vs. Malignant task do not indicate model failure. Training and validation curves for all architectures show stable convergence, confirming effective learning. Instead, the reduced performance reflects the intrinsic difficulty of this distinction, particularly for low-contrast or overlapping lesions, and is consistent with previous findings in both DM and CESM studies.

### 3.2. Model Interpretability

To better understand the decision-making process of the network, an interpretability analysis is conducted using SHAP on the best-performing architecture, EfficientNet-B0. This model is selected because it achieves the highest accuracy, precision, and AUC values across both DM and CESM modalities (Table 4 and Table 5). The objective is to visualize and quantify the image regions that most strongly influence model predictions, thereby providing an explainable perspective on how the network distinguishes between lesion types in both modalities.

**Figure 4:**
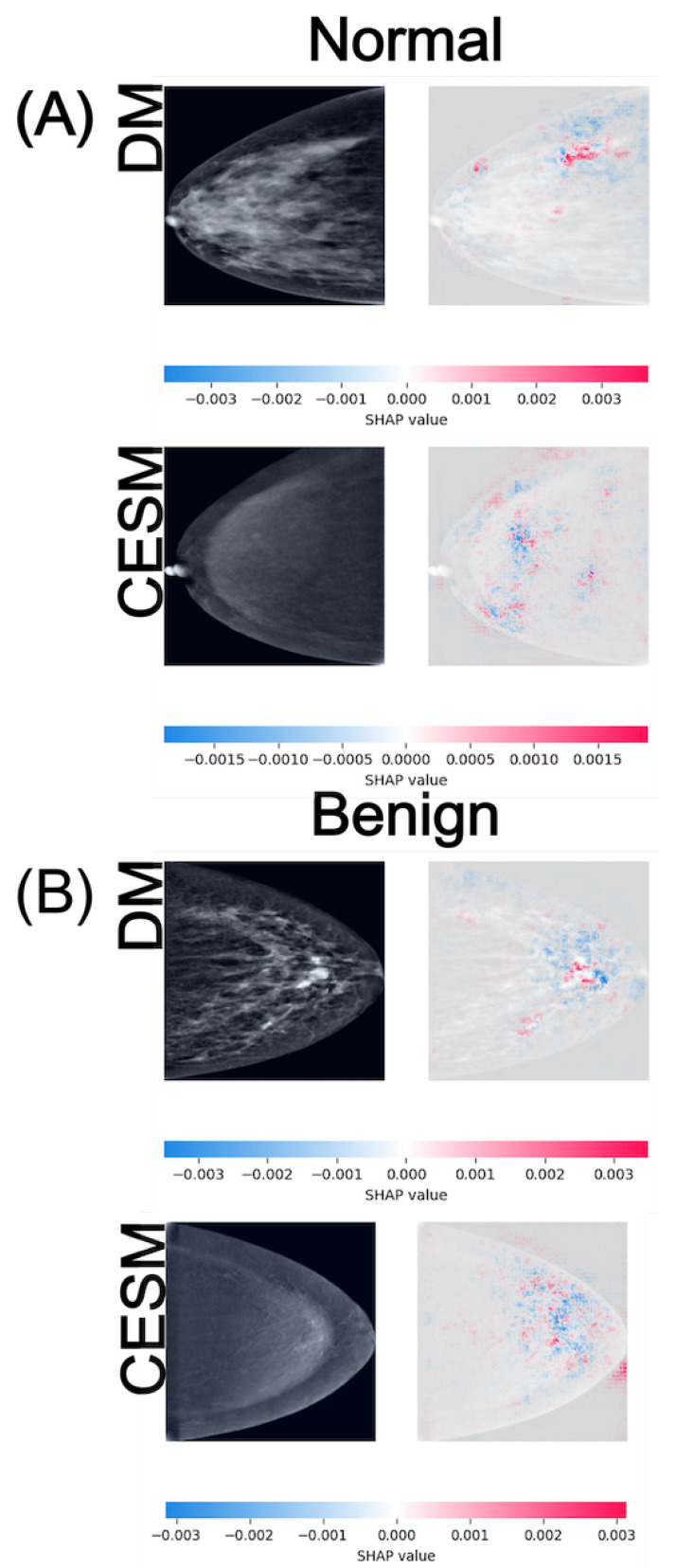
SHAP attribution maps for representative normal (**A**) and benign (**B**) cases in both DM and CESM using EfficientNet-B0. Each row corresponds to a different imaging modality (DM and CESM). The left panels show the original images, while the right panels display the SHAP overlays. The horizontal color bar below each row indicates the SHAP value scale, where blue represents negative contributions and red represents positive contributions to the predicted class.

**Figure 5:**
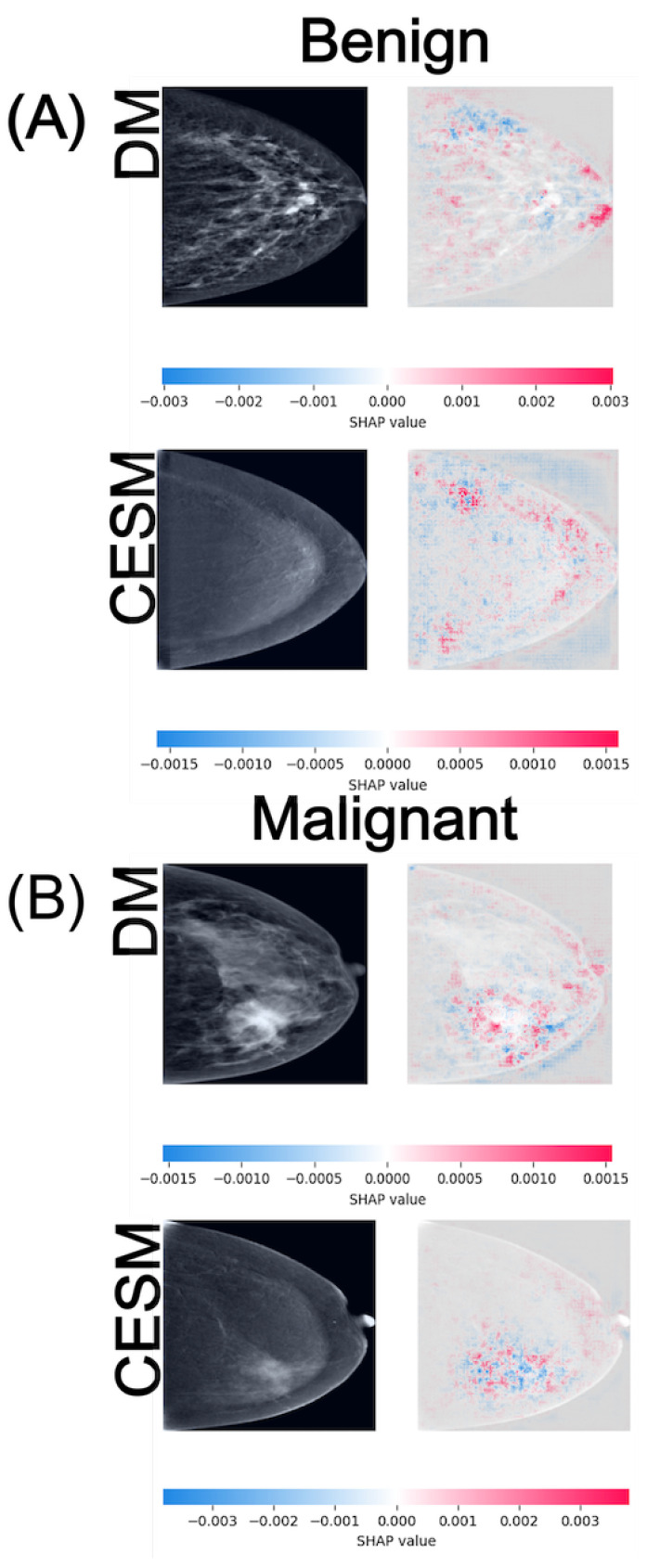
SHAP attribution maps for representative benign (**A**) and malignant (**B**) cases in both DM and CESM using EfficientNet-B0. Each row corresponds to a different imaging modality (DM and CESM). The left panels show the original images, while the right panels display the SHAP overlays. The horizontal color bar below each row indicates the SHAP value scale, where blue represents negative contributions and red represents positive contributions to the predicted class.

**Figure 6:**
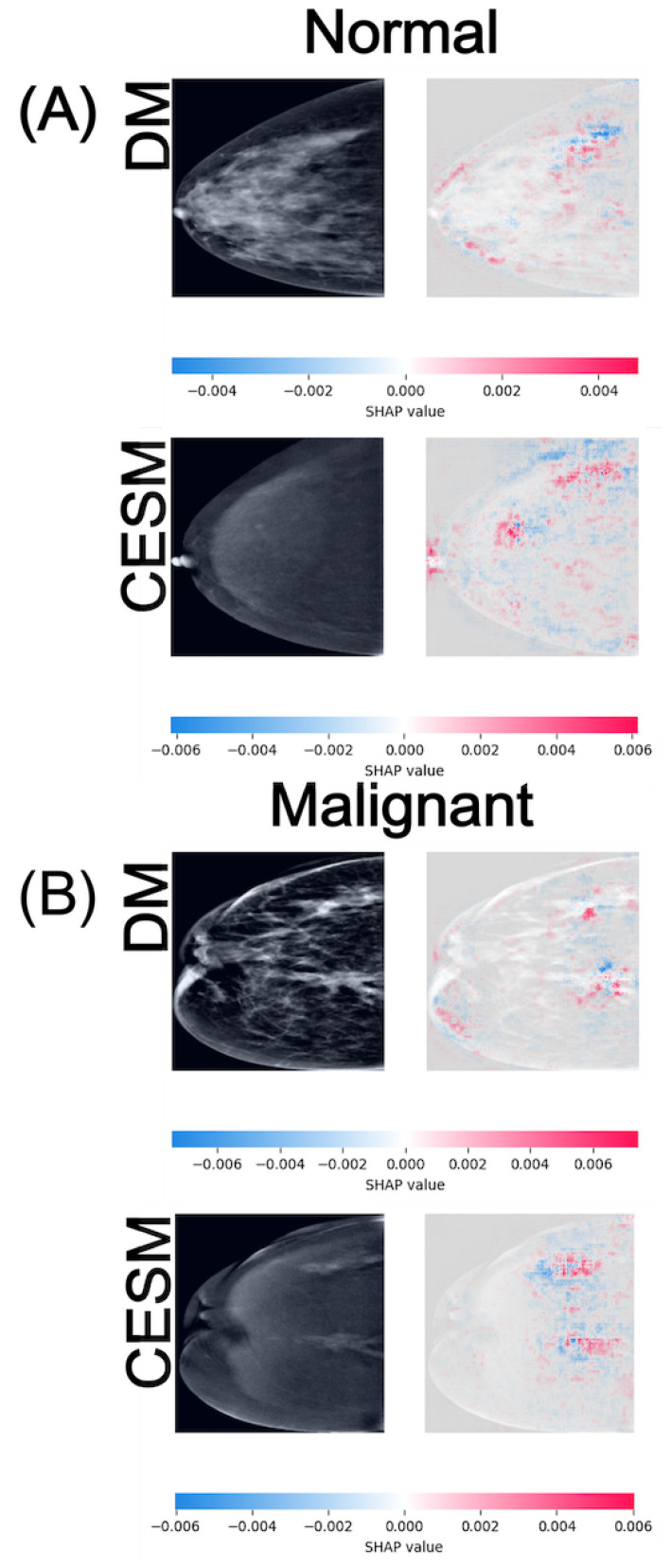
SHAP attribution maps for representative normal (**A**) and malignant (**B**) cases in both DM and CESM using EfficientNet-B0. Each row corresponds to a different imaging modality (DM and CESM). The left panels show the original images, while the right panels display the SHAP overlays. The horizontal color bar below each row indicates the SHAP value scale, where blue represents negative contributions and red represents positive contributions to the predicted class.

SHAP is employed as a game-theoretic framework that quantifies the contribution of each input feature to the model output. Pixel-wise SHAP values are computed for representative test samples using the GradientExplainer function, with the training set serving as the background distribution for expected output estimation. Positive SHAP values (red) denote regions that support the predicted class, whereas negative values (blue) indicate areas that counteract the prediction. These values are visualized as heatmaps superimposed on the original mammograms to facilitate anatomical interpretation. Figure 4, Figure 5 and Figure 6 illustrate representative examples for the three binary tasks: Normal vs. Benign, Benign vs. Malignant, and Normal vs. Malignant.

In the Normal vs. Benign task (Figure 4), both modalities exhibit anatomically consistent activation patterns. For normal cases (A), DM displays low-intensity SHAP values around ±0.003 distributed diffusely across the breast parenchyma, while CESM presents slightly lower magnitudes of approximately ±0.0015. These diffuse and low-amplitude activations are consistent with the homogeneous parenchymal density observed in both modalities, where no focal lesions or architectural distortions are present. For benign cases (B), DM shows SHAP activations around ±0.003 scattered near the lesion and its periphery, whereas CESM displays similar magnitudes but with more spatially concentrated contributions near regions of mild contrast enhancement. Despite minor amplitude differences, both modalities highlight comparable tissue patterns, indicating that DM preserves lesion-related cues even in the absence of contrast. The lesion is located in the lower-central quadrant of the breast, appearing as a small, well-circumscribed nodular opacity slightly inferior to the nipple line in DM, and as a mild, localized area of contrast uptake in CESM. In both modalities, SHAP activations cluster around this region, particularly along the lesion margins.

In the Benign vs. Malignant comparison (Figure 5), the SHAP maps reveal more distinct activation patterns across modalities. For benign cases (A), DM exhibits diffuse positive and negative contributions around ±0.003 distributed across the glandular tissue, whereas CESM shows lower-magnitude activations of approximately ±0.0015 with more localized distribution near areas of mild enhancement. In this benign example, the lesion is situated in the lower-outer quadrant. On DM, it appears as a round, smoothly marginated opacity, while CESM demonstrates minimal, non-focal enhancement in the corresponding region. SHAP contributions highlight this same area in both modalities, reflecting its limited but consistent influence on the benign classification. For malignant cases (B), DM shows moderate-intensity activations around ±0.0015 primarily outlining the lesion margins, whereas CESM produces stronger and more spatially concentrated contributions near enhancing tumor regions, reaching values of approximately ±0.003. Although CESM provides higher spatial precision, DM still captures lesion-related regions consistent with structural irregularities, indicating preservation of key diagnostic cues. In this malignant example, the tumor is located in the lower-inner quadrant. In DM, it appears as a dense mass with irregular borders and associated parenchymal distortion, while CESM shows intense, focal contrast uptake at the same location. SHAP maps densely highlight this region in both modalities, aligning with the tumor boundaries and the areas of strongest enhancement.

In the Normal vs. Malignant task (Figure 6), the SHAP maps exhibit the widest value ranges among all comparisons, reflecting the increased complexity of distinguishing malignant lesions from normal tissue. For normal cases (A), DM activations are diffuse and texture-driven, with SHAP magnitudes around ±0.004, whereas CESM presents slightly higher and more structured attributions centered on subtle enhancement patterns, reaching ±0.006. In this normal example, the breast shows no suspicious focal abnormalities. The parenchymal pattern is uniformly distributed, resulting in scattered, low-magnitude SHAP activations without a dominant region of interest. For malignant cases (B), both modalities produce strong positive SHAP contributions within the lesion region, with DM and CESM reaching similar maximum values of approximately ±0.006. These broader SHAP ranges indicate greater model sensitivity to discriminative features associated with malignancy. Despite minor quantitative differences, both modalities highlight spatially aligned hotspots, suggesting that DM and CESM emphasize analogous anatomical regions when identifying cancer. In this malignant example, the lesion is located in the upper-outer quadrant. The DM image reveals a high-density mass with irregular margins, while CESM demonstrates strong, well-localized contrast uptake in the corresponding area. SHAP hotspots converge on this region in both modalities, indicating high model confidence in detecting malignant features.

Taken together, these results demonstrate that although CESM provides more spatially concentrated activations due to its functional contrast component, DM also exhibits consistent and interpretable SHAP patterns that align with the same underlying anatomical structures. This convergence suggests that the discriminative visual cues leveraged by the network are shared between modalities, indicating that conventional DM, despite its lower numerical performance, still provides meaningful diagnostic information explainable by AI models. Such findings reinforce the value of DM as a feasible alternative in environments where CESM is unavailable, promoting equitable access to explainable, AI-assisted breast cancer screening.

Taken collectively, the spatial organization, polarity, and intensity of SHAP activations provide an additional perspective on model certainty. Focal, well-delimited hotspots reflect higher discriminative confidence, whereas diffuse or inconsistent patterns indicate ambiguity or limited feature separability. Evaluating these spatial cues therefore helps clarify not only the outcome of each prediction but also the relative difficulty of the underlying diagnostic task.

Overall, the SHAP visualizations corroborate the quantitative metrics reported earlier. Tasks with higher AUC values, such as Normal vs. Malignant, exhibit more distinct and spatially coherent activations, indicating that increased model certainty corresponds to more anatomically focused explanations. Conversely, in the Normal vs. Benign task, where the AUC approaches random performance, SHAP maps display dispersed and inconsistent patterns, reflecting uncertainty in the learned features. These observations highlight the interpretive value of SHAP in assessing not only what the model predicts but also how prediction confidence varies across modalities and diagnostic categories.

## 4. Discussion

This study presents a comprehensive comparison of three CNN architectures, ResNet-18, DenseNet-121, and EfficientNet-B0, for breast lesion classification using DM and CESM. The results reveal clear performance differences between models and modalities, while SHAP-based interpretability analysis provides valuable insight into the spatial reasoning underlying CNN decisions. Together, these findings offer a balanced understanding of how model architecture and imaging modality jointly influence diagnostic performance and clinical reliability.

Across all metrics, CESM consistently outperforms DM in tasks requiring the discrimination of subtle tissue differences, such as Normal vs. Benign and Benign vs. Malignant. The use of iodine-based contrast improves lesion conspicuity and enhances discriminative power, reflected in higher AUC, precision, and F1-scores across architectures. In contrast, DM demonstrates stronger separability in the Normal vs. Malignant task, suggesting that structural and textural information alone can be sufficient for identifying overt malignancies. These results underscore the complementary diagnostic value of both modalities: CESM provides functional information that facilitates early and accurate characterization of complex lesions [42,43], whereas DM remains a reliable and interpretable baseline for broad screening workflows [44,45].

Although EfficientNet-B0 is selected as the reference model due to its overall stability and superior performance across modalities, its AUC in the Normal vs. Benign task for DM approximates random classification, indicating limited discriminative learning in this specific scenario. This behavior is visually supported by the SHAP attribution maps (Figure 4), where activations appear spatially inconsistent compared to the other tasks. Such diffusion likely reflects stochastic learning rather than meaningful lesion recognition. In contrast, SHAP visualizations for the Benign vs. Malignant and Normal vs. Malignant tasks display anatomically coherent attention patterns across both modalities. CESM heatmaps show focal attributions concentrated on contrast-enhancing tissue, whereas DM maps, although more diffuse, consistently highlight the same anatomical regions. This convergence supports the reliability of DM-based representations, even when the absence of contrast reduces localization sharpness.

Particularly noteworthy is the performance of DM in the Normal vs. Malignant comparison, where the AUC surpasses that of CESM. This finding is clinically significant because DM is more widely available and less resource-intensive than CESM, making it a cornerstone modality in low- and middle-income healthcare systems. The ability of DM to achieve high separability between malignant and normal cases demonstrates that conventional mammography, when analyzed using well-tuned CNNs, provides accurate and interpretable diagnostic cues. This strengthens its role in accessible AI-assisted breast cancer screening. Moreover, such performance aligns with clinical screening priorities, where differentiating malignant from normal cases is critical for triage and early intervention, even without contrast enhancement.

When comparing CNN architectures, EfficientNet-B0 achieves the most balanced and superior performance across modalities, particularly in test AUC and precision. Its compound scaling strategy and efficient parameter utilization enable it to capture richer hierarchical representations without overfitting. DenseNet-121, while occasionally achieving competitive results during cross-validation, shows greater variability in the test set, suggesting increased sensitivity to distribution shifts. ResNet-18 demonstrates stable but conservative behavior, producing moderate F1-scores and recall values that reflect robustness but limited discriminative capacity in complex cases. These architectural differences highlight the importance of depth, connectivity, and representational efficiency in capturing multimodality imaging features. Recent studies further emphasize the continued competitiveness of EfficientNet-based models in medical imaging, even relative to emerging transformer architectures, owing to their parameter efficiency and stable performance on small to medium-sized datasets.

To contextualize these findings within existing literature, Table 6 summarizes selected deep learning studies that address breast lesion classification using DM and CESM. Although methodological details and target tasks vary among studies, the reported results collectively establish reference benchmarks for CNN-based mammographic analysis.

In comparison with prior research, our results show that EfficientNet-B0 achieves competitive or superior discriminative performance across modalities. For instance, Aboutalib et al. (2018) [46] report AUC values between 0.76 and 0.91 for distinguishing benign from malignant findings in DM without contrast, whereas our EfficientNet-B0 model attains an AUC of 0.97 in the Normal vs. Malignant task using DM, representing a substantial improvement in classification separability. This highlights the ability of modern architectures to extract richer morphological features even from non-contrast data. Similarly, Ribli et al. (2018) [47] achieve an AUC of approximately 0.85 using a Faster R-CNN approach on the INbreast dataset, comparable to our CESM results, which exceed 0.93 in the same task.

Moreover, Helal et al. (2024) [48] validate a multiview deep-learning framework on CESM that achieves AUC values between 0.90 and 0.94 for benign versus malignant classification, supporting the high discriminative capacity of CNNs when contrast enhancement is available. Their findings are consistent with our CESM results, reinforcing that contrast information enhances lesion separability through improved vascular and morphological characterization.

Finally, Qasrawi et al. (2024) [49] demonstrate that ensemble CNNs can achieve high accuracy (96.6%) on DM, consistent with our observation that EfficientNet-B0 effectively leverages structural and textural cues even without contrast information. This comparison underscores that, while prior CNN-based systems report strong performance on individual datasets, our framework extends this evidence by directly contrasting CESM and DM under identical preprocessing, training, and evaluation conditions, thereby providing a controlled benchmark for cross-modality generalization.

Unlike previous studies, which typically evaluate either DM or CESM independently, the present work directly compares both modalities under identical preprocessing, training, and evaluation conditions, thereby providing a harmonized framework that strengthens the validity of cross-modality conclusions and highlights the novelty of this approach.

It is important to note that the studies summarized in Table 6 do not use the same dataset employed in the present work. None of the prior publications were conducted using the CDD-CESM dataset, nor did they evaluate DM and CESM under a harmonized pipeline with identical preprocessing, training, and evaluation criteria. Therefore, the values reported in Table 6 serve as contextual benchmarks rather than direct numerical comparisons. A key contribution of this study is that, to the best of our knowledge, it is the first to analyze both DM and CESM within the same dataset and modeling framework, enabling a controlled modality-level comparison that is not available in prior literature.

The interpretability results derived from SHAP further contextualize these quantitative findings. In both DM and CESM, the CNNs attend to anatomically coherent regions, primarily surrounding lesions or areas of contrast uptake. CESM produces sharper and more localized SHAP activations, consistent with contrast-driven delineation of vascularized tissue, whereas DM yields broader but still meaningful attention patterns focused on parenchymal structures. This alignment between modalities indicates that the models learn semantically relevant visual cues regardless of contrast enhancement. These consistent attribution patterns across modalities support the integration of SHAP-based interpretability as a quality-control step in clinical AI pipelines, ensuring that model predictions remain anatomically and pathophysiologically meaningful.

Benign lesion classification remains the most challenging task across all models and modalities. SHAP maps reveal dispersed and inconsistent attributions, suggesting model uncertainty in differentiating benign from malignant or normal tissue patterns. In CESM, mild enhancement occasionally leads to overestimation of malignancy likelihood, whereas in DM, low-contrast lesions are often underrepresented. These observations indicate that even state-of-the-art CNNs struggle to capture the nuanced imaging characteristics of benign lesions. Integrating radiologist-annotated regions of interest, fine-tuning with domain-specific loss functions, or adopting attention-based or transformer architectures could help refine decision boundaries and improve clinical reliability in this category.

The consistent spatial overlap observed between DM and CESM SHAP maps is an encouraging finding for clinical adoption. It implies that, despite quantitative performance differences, the networks base their predictions on biologically plausible and clinically relevant features. This spatial agreement across modalities strengthens trust in AI-assisted systems, supporting their use as complementary diagnostic tools rather than opaque classifiers. Furthermore, the convergence of model attention across architectures suggests that learned representations are not arbitrary but anchored to consistent anatomical cues.

A brief SWOT perspective further contextualizes these findings. The main strengths of this work include the harmonized DM–CESM preprocessing pipeline and the use of SHAP for transparent interpretability across models and modalities. A key weakness is the reliance on a single public dataset, which may limit generalizability. Opportunities arise from extending the framework to multiclass analyses, incorporating larger multi-institutional datasets, and integrating transformer-based architectures. Potential threats include variability in CESM acquisition protocols and the risk that modality-specific artifacts may influence model predictions if not carefully controlled.

From a clinical perspective, the three binary models evaluated in this study could be integrated into a sequential decision-making workflow. A Normal vs. Malignant classifier may function as an initial triage tool, identifying cases that require further diagnostic evaluation. Images classified as benign or suspicious could then be analyzed using the Benign vs. Malignant model to refine the decision and reduce unnecessary biopsies. This cascaded approach allows each model to operate within the diagnostic boundaries for which it is most reliable. Although a full multi-stage deployment system is beyond the scope of the present study, preliminary evaluation of such a combined workflow suggests that overall diagnostic accuracy would depend on the cumulative performance of each model in its respective decision step.

Despite the encouraging results, several limitations should be acknowledged. The dataset size, although balanced across classes, restricts generalizability and may amplify model variance, particularly in the benign category. The use of JPEG-compressed images instead of raw DICOM data may also reduce preservation of fine-grained texture features critical for subtle lesion discrimination. Moreover, the evaluation focuses on three architectures; expanding future analyses to include transformer-based or hybrid CNN–Transformer models could offer a more comprehensive understanding of feature abstraction in breast imaging. Finally, the SHAP analysis, while effective for qualitative interpretation, remains computationally expensive and may not fully capture complex nonlinear dependencies across feature hierarchies.

Collectively, the results demonstrate that CESM enhances classification performance through functional contrast information, whereas DM retains strong interpretability and clinical relevance. EfficientNet-B0 emerges as the most effective and explainable architecture, providing stable and anatomically coherent attributions across modalities. These insights reinforce the potential of explainable deep learning frameworks to bridge diagnostic accuracy with transparency, ultimately supporting equitable and reliable AI integration into breast cancer screening and diagnostic workflows. Future extensions of this framework could include multi-center datasets and multimodal fusion approaches, enabling a more holistic understanding of lesion behavior across imaging modalities and populations.

## 5. Conclusions

This study presents a comprehensive comparison of CNN-based models for breast lesion classification using DM and CESM. The findings confirm that CESM enhances diagnostic performance across most evaluation metrics due to the addition of functional contrast information. However, DM maintains strong predictive capability and interpretability, particularly in the Normal vs. Malignant task, where it achieves a higher AUC than CESM. This result underscores the continued clinical relevance of DM, which remains the most accessible imaging modality for population-level screening, especially in resource-limited settings.

The analysis of SHAP attribution maps further reinforces these conclusions by revealing that both modalities focus on comparable anatomical regions associated with lesion presence. CESM produces more spatially concentrated attributions, whereas DM generates broader yet anatomically consistent activation patterns. These findings demonstrate that even without contrast enhancement, DM-based deep learning models capture meaningful diagnostic cues that remain clinically coherent and interpretable.

EfficientNet-B0 emerges as the most effective architecture across both modalities, showing stable performance and anatomically coherent SHAP distributions. Nevertheless, certain tasks, particularly Normal vs. Benign in DM, remain challenging due to limited discriminative information and stochastic learning effects.

Overall, this study highlights the potential of explainable AI to enhance breast cancer detection by combining quantitative performance with clinical transparency. The results support the continued optimization and deployment of DM-based models as equitable, accessible, and explainable tools within AI-assisted breast imaging workflows.

## Figures and Tables

**Table 1:** Distribution of images per diagnostic class and modality in the CDD-CESM dataset [32].

Class	DM Images	CESM Images	Total
Normal	341	416	757
Benign	331	256	587
Malignant	331	331	662
**Total**	**1003**	**1003**	**2006**

**Table 2:** Preprocessing and augmentation transformations applied to training images, and deterministic preprocessing used for validation and test sets.

Operation	Description	Parameters
Resize	Resizes all images to a uniform input size	(224, 224)
RandomHorizontalFlip	Randomly flips the image horizontally	p=0.5
RandomVerticalFlip	Randomly flips the image vertically	p=0.5
RandomRotation	Applies small in-plane rotations to improve generalization	$\pm 20^{\circ }$
ColorJitter	Randomly alters brightness and contrast	brightness = 0.05; contrast = 0.15
ToTensor	Converts the image to a tensor format	–
Normalize	Normalizes pixel values using ImageNet statistics	mean = [0.485, 0.456, 0.406]; std = [0.229, 0.224, 0.225]
Validation and test: Resize (224, 224) and Normalize (ImageNet mean/std).		

**Table 3:** Summary of model architecture and training configuration.

Configuration	Value
Architectures	ResNet-18, DenseNet-121, EfficientNet-B0 (pre-trained on ImageNet)
Loss function	Weighted BCE (inverse class frequency)
Optimizer	Adam
Initial learning rate	$1\times 10^{-3}$
Learning rate scheduler	StepLR (step size = 10, γ=0.1)
Batch size	8
Epochs	30
Cross-validation	3-fold (training only)
Test set	20% independent, patient-level split
Output activation	Sigmoid (single-logit output)

**Table 4:** Performance metrics for DM (binary classification results of ResNet-18, DenseNet-121, and EfficientNet-B0).

Task	Model	Cross-Validation (CV)					Test Set				
		ACC (%)	PREC (%)	REC (%)	F1 (%)	AUC (%)	ACC (%)	PREC (%)	REC (%)	F1 (%)	AUC (%)
**Normal vs. Benign**	ResNet-18	49.83	50.52	38.88	43.87	50.67	50.00	38.89	56.00	45.90	54.44
	DenseNet-121	50.83	51.21	47.54	49.16	52.91	37.88	30.00	48.00	36.92	44.88
	**EfficientNet-B0**	**62.38**	**66.36**	**53.32**	**58.63**	**67.02**	**54.55**	**43.59**	**68.00**	**53.13**	**57.66**
**Benign vs. Malignant**	ResNet-18	64.90	65.17	62.50	63.61	70.68	49.12	56.52	40.63	47.27	58.50
	DenseNet-121	63.24	63.05	63.33	62.76	68.63	57.89	65.38	53.13	58.62	65.50
	**EfficientNet-B0**	**68.38**	**68.73**	**67.12**	**67.56**	**76.06**	**63.16**	**73.91**	**53.13**	**61.82**	**80.75**
**Normal vs. Malignant**	ResNet-18	76.09	77.36	73.77	75.50	83.74	67.13	63.33	59.38	61.29	77.51
	DenseNet-121	74.42	75.26	73.07	74.07	82.43	68.49	66.67	56.25	61.02	75.46
	**EfficientNet-B0**	**82.11**	**87.97**	**74.67**	**80.50**	**89.48**	**93.15**	**93.54**	**90.63**	**92.06**	**97.33**

**Table 5:** Performance metrics for CESM (binary classification results of ResNet-18, DenseNet-121, and EfficientNet-B0).

Task	Model	Cross-Validation (CV)					Test Set				
		ACC (%)	PREC (%)	REC (%)	F1 (%)	AUC (%)	ACC (%)	PREC (%)	REC (%)	F1 (%)	AUC (%)
**Normal vs. Benign**	ResNet-18	67.19	69.43	62.08	65.00	71.05	59.09	47.37	72.00	57.14	62.05
	DenseNet-121	70.90	72.43	69.17	70.37	78.33	60.61	47.83	44.00	45.83	55.90
	**EfficientNet-B0**	**81.32**	**84.15**	**79.00**	**81.39**	**86.49**	**77.27**	**70.83**	**68.00**	**69.39**	**84.68**
**Benign vs. Malignant**	ResNet-18	68.02	70.18	66.67	68.01	76.24	65.52	72.41	63.64	67.74	72.00
	DenseNet-121	71.69	73.49	70.04	71.66	80.03	70.69	75.00	72.73	73.85	78.55
	**EfficientNet-B0**	**76.32**	**77.41**	**75.22**	**76.16**	**82.08**	**72.41**	**81.48**	**66.67**	**73.33**	**74.30**
**Normal vs. Malignant**	ResNet-18	82.43	84.10	80.73	82.38	88.43	86.46	84.85	84.85	84.85	91.28
	DenseNet-121	83.53	85.21	81.84	83.38	89.72	89.19	90.32	84.85	87.50	93.87
	**EfficientNet-B0**	**85.66**	**88.75**	**82.56**	**85.54**	**91.00**	**87.88**	**83.33**	**90.91**	**86.96**	**93.27**

**Table 6:** Selected comparative studies in breast lesion classification using deep learning (DL) on Digital Mammography (DM) and Contrast-Enhanced Spectral Mammography (CESM).

Study (Year)	Modality	Architecture	Task	AUC/ACC %
Aboutalib et al., 2018 [46]	DM	CNN (custom)	Benign vs. Malignant	AUC = 76 to 91
Ribli et al., 2018 [47]	DM	Faster R-CNN	Mass Detection/Classification	AUC = 85
Dominique et al., 2022 [26]	CESM	CheXNet-based CNN	ER status/Triple-negative	AUC = 83 to 91
Helal et al., 2024 [48]	CESM	Deep CNN (multiview)	Benign vs. Malignant	AUC = 90 to 94
Qasrawi et al., 2023 [49]	DM	Ensemble (CNN)	Benign vs. Malignant	ACC = 96.6
**This study (2025)**	DM	EfficientNet-B0 (fine-tuned)	Normal vs. Malignant	AUC = 97
**This study (2025)**	CESM	EfficientNet-B0 (fine-tuned)	Normal vs. Malignant	AUC = 93

Note: Performance values for this study correspond to test AUC results reported in Table 4 and Table 5.

## Data Availability

Data are available at: https://github.com/omar-mohamed/CDD-CESM-Dataset (accessed on 1 April 2025).

## Abbreviations

The following abbreviations are used in this manuscript:

ACC	Accuracy
AI	Artificial Intelligence
AUC	Area Under the Curve
BCE	Binary Cross-Entropy
CC	Craniocaudal
CESM	Contrast-Enhanced Spectral Mammography
CNN	Convolutional Neural Network
DL	Deep Learning
DM	Digital Mammography
F1	F1-Score (Harmonic Mean of Precision and Recall)
FN	False Negative
FP	False Positive
LE	Low-Energy (DM-equivalent image in CESM)
MLO	Mediolateral Oblique
MRI	Magnetic Resonance Imaging
PREC	Precision
REC	Recall (Sensitivity)
ROC	Receiver Operating Characteristic
SHAP	SHapley Additive exPlanations
TN	True Negative
TP	True Positive
XAI	Explainable AI
